# Report of Nine Months' Work

**Published:** 1920-11-27

**Authors:** 


### LEICESTER ROYAL INFIRMARY.

### Report of Nine Months' Work.

The growing demands upon voluntary hospitals were evidenced by the statistics submitted to the quarterly meeting of the Leicester Royal Infirmary.

In-patients numbered 3,312, an increase of 70. The average daily number of beds occupied was 264, against 244. Out-patients' attendances were 34,683, against 26,059. Casualties 51,467, against 41,120. X-ray cases 3,502, against 3,311. Operations 4,409, against 3,793. 7,069 reports were made by the pathological department, compared with 4,204. Attendances in the new orthopædic department numbered 11,710, and the treatments 15,384.

The finances reflected the fact that the increase of patients in all departments, together with the development of the institution in the equipment of a new orthopædic department and a new pathological laboratory, had synchronised with a period in which expenses had been abnormally high. The institution and the medical staff had, however, given unstinted service.

The available income for the period was £36,120, the expenditure £48,007. A great advance was noted in the income from donations, £5,755, as compared with £3,721 in the corresponding period. This was largely due to the number of village fêtes, vegetable shows, whist drives, etc., which had been organised.

Investments produced £2,882, an increase of £450. The Saturday Hospital Fund was £9,533, as compared with £4,853, reflecting the increase in the contributions from 1d. to 2d. per week. The income of the children's hospital was £3,005, compared with £1,400. £4,545 had been received in legacies, £2,700 of which was for bed endowments.

### Cost of Supplies Greatly Increased.

Expenditure had increased at an alarming rate. Provisions at £10,680 showed an advance of £1,500. Surgery and dispensary at £6,565, an increase of £2,000, due to large purchases in the early months of the year, the advantage of which would not be observable until next year. Domestic expenses have increased to £9,158, £2,500 more than in the previous year. Municipal charges for gas and electric-light supplies and fuel and lighting largely accounted for this increase. The water account showed an increase of 50 per cent. on the figures for 1914, and gas and electric current an increase of 60 per cent. Coal and coke had advanced from £1,370 in 1914 to £4,000.

Salaries and wages, comparing 1920 with 1919, advanced to £16,000 from £11,000, making a total increase in expenditure of £12,000 on the nine months' figures.

Considerable building operations have been in progress with a view to developing the institution to meet increasing needs; sixty-six bedrooms were being added to the nurses' home, at an approximate cost of £20,000. Furnishing will cost £60 per room—a price which compared with £15 per room when the home was first furnished.

Excavations for a new west wing of a hundred beds were now being undertaken; the cost of the new wing would be £35,000, with a further £10,000 for furnishing. Towards these extensions £42,000 had been raised. The upkeep of these new additions was the cause of the warning note which the Board sounded with regard to the income of the future.

### 150th Anniversary.

The Board hoped that the commemoration of the 150th Anniversary next year would have been an ideal opportunity to increase the income of the institution, but commercial inactivity, the uncertainty everywhere apparent, and the industrial unrest created obscurity and made it very difficult to look ahead.

To be successful the organisation should have been commenced; but if the clouds that overshadowed the nation should rise within the next few months, as surely might be hoped, it would be possible to undertake a modified effort to provide ways and means for the maintenance of a service manifestly essential to the public well-being.

